# Benchmark of computational methods for predicting microRNA-disease associations

**DOI:** 10.1186/s13059-019-1811-3

**Published:** 2019-10-08

**Authors:** Zhou Huang, Leibo Liu, Yuanxu Gao, Jiangcheng Shi, Qinghua Cui, Jianwei Li, Yuan Zhou

**Affiliations:** 1 Department of Biomedical Informatics, Department of Physiology and Pathophysiology, Center for Noncoding RNA Medicine, MOE Key Lab of Cardiovascular Sciences, School of Basic Medical Sciences, Peking University, 38 Xueyuan Rd, Beijing, 100191 China; 2 Institute of Computational Medicine, School of Artificial Intelligence, Hebei University of Technology, Tianjin, 300401 China; 3 Center of Bioinformatics, Key Laboratory for Neuro-Information of Ministry of Education, School of Life Science and Technology, University of Electronic Science and Technology of China, Chengdu, 610054 China

**Keywords:** Benchmarking test, miRNA-disease association, Disease miRNA prediction

## Abstract

**Background:**

A series of miRNA-disease association prediction methods have been proposed to prioritize potential disease-associated miRNAs. Independent benchmarking of these methods is warranted to assess their effectiveness and robustness.

**Results:**

Based on more than 8000 novel miRNA-disease associations from the latest HMDD v3.1 database, we perform systematic comparison among 36 readily available prediction methods. Their overall performances are evaluated with rigorous precision-recall curve analysis, where 13 methods show acceptable accuracy (AUPRC > 0.200) while the top two methods achieve a promising AUPRC over 0.300, and most of these methods are also highly ranked when considering only the causal miRNA-disease associations as the positive samples. The potential of performance improvement is demonstrated by combining different predictors or adopting a more updated miRNA similarity matrix, which would result in up to 16% and 46% of AUPRC augmentations compared to the best single predictor and the predictors using the previous similarity matrix, respectively. Our analysis suggests a common issue of the available methods, which is that the prediction results are severely biased toward well-annotated diseases with many associated miRNAs known and cannot further stratify the positive samples by discriminating the causal miRNA-disease associations from the general miRNA-disease associations.

**Conclusion:**

Our benchmarking results not only provide a reference for biomedical researchers to choose appropriate miRNA-disease association predictors for their purpose, but also suggest the future directions for the development of more robust miRNA-disease association predictors.

## Introduction

MicroRNAs (miRNAs) are ~ 22 nt RNAs that regulate gene expression mainly by targeting the 3′UTR regions of mRNAs [1, 2]. These small non-coding RNAs are widely involved in important biological processes such as cell division, differentiation, apoptosis, cell cycle regulation, inflammation, and stress response [3, 4]. Therefore, dysregulations of miRNAs, including expression de-regulation, gain- or loss-of-function mutation, and epigenetic silencing, often play important roles in the onset and development of many diseases including but not limited to cancer, cardiovascular diseases, and neurodegenerative diseases [5–7]. To date, there are a few popular databases of miRNA-disease associations, among which HMDD and miR2Disease manually curate known miRNA-disease associations from literature, while dbDEMC infers miRNA-disease associations by identifying the differentially expressed miRNAs in disease conditions (cancers) observed in public transcriptome datasets [8–11]. These databases could be used not only for biomedical scientists to understand the roles of miRNAs in diseases, but also for bioinformatics developers to establish novel miRNA-disease association prediction tools. Indeed, given that the large proportion of potential miRNA-disease associations remain unexplored, the computational approaches constitute an essential complement to the experimental assays. For instance, the latest miRBase (v22.1, October 2018) has recorded 1917 human miRNA genes [12], while there are more than 9000 disease terms according to the current Disease Ontology (DO) nomenclature [13]. By contrast, HMDD v3.1, the most updated miRNA-disease association dataset for now (released in January 2019), covers only 35,547 miRNA-disease associations between 893 diseases and 1206 miRNA genes [8]. These statistics indicate that ~ 30% and ~ 80% of human miRNAs and diseases respectively have not been reported by experimental investigations. Considering the time and labor cost of experimental assays, efficient and accurate computational prediction tools are necessary and warranted for the community to screen primary targets for further studies.

To this end, novel prediction methods for miRNA-disease associations have been continuously proposed. These methods can be largely grouped into three categories: (1) methods based on score function, (2) methods based on the complex network or graph algorithms, and (3) methods based on the machine learning algorithms [14]. By assuming that functional-related miRNAs are more likely to be associated with phenotypically similar diseases, the first category of methods designed various scoring functions to estimate the functional similarity between miRNAs. One early method developed a scoring system assuming that the microRNA pairs linked to common diseases were functionally more related [6]. More sophisticated scoring functions can be constructed by extracting scoring terms from the miRNA-miRNA and disease-disease networks. For example, WBSMDA integrated features from miRNA functional similarity network, disease semantic similarity network, and Gaussian interaction profile kernel similarity network to infer the potential disease-miRNA associations [11]. The network or graph algorithms focused on constructing miRNAs and/or disease similarity networks and efficient transferring miRNA-disease association labels between similar miRNAs and/or similar diseases in the network. Therefore, label propagation algorithm, which has the advantages of simplicity and efficiency on the miRNA/disease similarity networks, often constitutes the core component of the algorithm framework for this type of methods, e.g., MCLPMDA [15], LPLNS [16], SNMDA [17], and HLPMDA [18]. Nevertheless, more sophisticated algorithm designs are often crucial for successful prediction of miRNA-disease associations. For example, MCLPMDA employed matrix completion algorithm in addition to label propagation, LPLNS adopted linear neighborhood similarity when implementing label propagation, SNMDA introduced sparse neighborhood representation for building the similarity network, and HLPMDA took a heterogeneous label propagation approach to transfer association label among a heterogeneous set of similarity networks [15–18]. Other algorithms focusing on the specific topology of miRNA-disease association network have also been proposed, such as BNPMDA [19] that used the bipartite network projection and SACMDA [20] that made predictions with short acyclic connections in a heterogeneous graph. On the other hand, machine learning classification algorithm could take advantages of the inherent features of miRNAs and diseases, or using the state-of-the-art recommendation algorithms therefore could also achieve a satisfactory performance. For example, as the first model using decision tree learning, EGBMMDA has reported a global leave-one-out cross-validation (LOOCV) area under ROC curve (AUROC) greater than 0.9 [21]. And other machine learning algorithms, such as collaborative filtering adopted by ICFMDA [22] and latent feature extraction with positive samples taken by LFEMDA [23], also showed promising performances in cross-validation tests.

Nevertheless, one emerging critical issue for these algorithms turns out to be the lack of an independent benchmarking test. According to our survey on PubMed and Google Scholar references, there are more than 100 articles describing 90 miRNA-disease association prediction methods, among which 36 tools are readily available as either source code or pre-calculated prediction results (Additional file 1: Table S1). Most of these methods used HMDD v2.0 data [24] as their training dataset and performed cross-validation test (either five- or tenfold cross-validation or LOOCV) on this dataset. While cross-validation is generally acceptable for performance assessment, the robustness of the prediction model on novel data and the risk for over-fitting to the training samples cannot be sufficiently assessed by cross-validation. This problem has become even more prominent now, since the HMDD v2.0 dataset was released 5 years ago, and a considerable amount of novel miRNA-disease associations have been reported in recent publications, making the previous HMDD v2.0 dataset less representative to the latest knowledge about miRNA-disease associations. As a compromise, developers of the prediction tools could also collect novel miRNA-disease associations from other databases or literature. However, since the manual literature curation is a labor-intensive task and requires specific biomedical background knowledge, the collected new associations were limited to few diseases or miRNAs and therefore could not constitute a sizable and qualified independent benchmarking dataset.

Recently, we have launched the updated HMDD v3.0 miRNA-disease association database [8], and as previously mentioned, its 3.1 version covers 35,547 miRNA-disease associations, which indicates more than threefold association data compared to the previous HMDD v2.0 (10,381 associations). This new dataset predisposes an unprecedented opportunity to benchmark the current prediction methods. Therefore, in this study, based on the novel miRNA-disease associations in HMDD v3.1, we have performed a comprehensive assessment of 36 readily available prediction methods [15–23, 25–51] from five aspects: First, we tested the overall performance of these methods by rigorous precision-recall curve analysis. Second, we assessed the mutual complementarity of these methods by iteratively combining the top-ranked methods for a better performance. Third, we checked if the overrepresentation of few miRNAs and diseases in current miRNA-disease association data would result in biased prediction results. Fourth, as many methods work with miRNA similarity data, we evaluated the influence of the updated miRNA similarity data by replacing the previous MISIM v1.0 miRNA similarity matrix [52] with the recently published MISIM v2.0 matrix (which was built on HMDD v3.0 data) [53]. Finally, identifying the disease causal miRNAs is of particular importance for medical researches on the disease mechanism and for identifying target miRNAs for further interventions. In the last update of HMDD (v3.2), although no new miRNA-disease association data was added, we systematically re-evaluated the causality potentials of the miRNAs to the corresponding diseases. Taking this opportunity, we also interrogated whether current prediction methods, which aimed at predicting general disease-associated miRNAs, could also prioritize the disease causal miRNAs.

## Results and discussion

### Independent benchmarking of miRNA-disease association prediction methods on novel HMDD v3.1 data

**Fig. 1:**
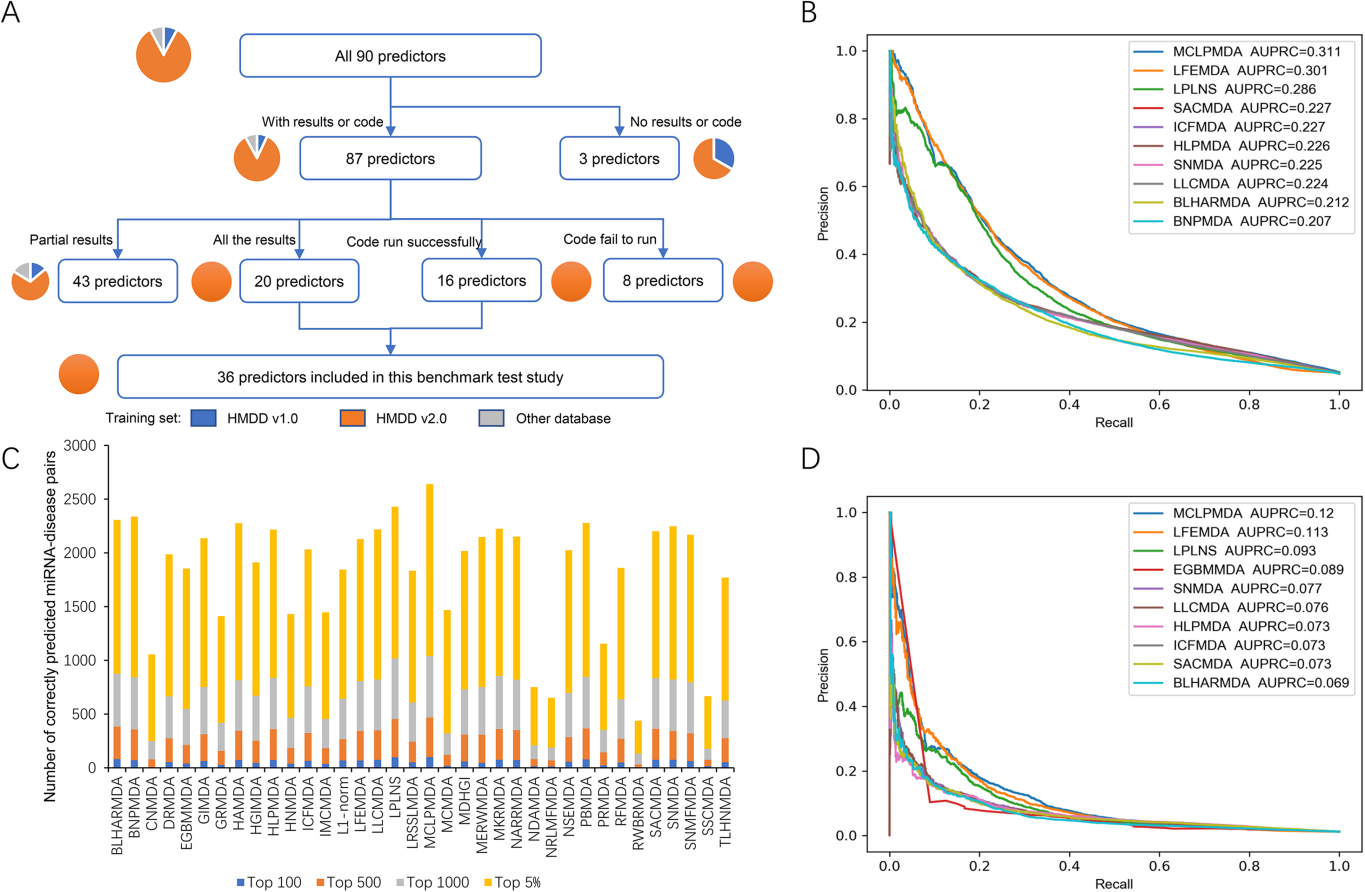
Overall performance of 36 miRNA-disease association predictors on the benchmarking datasets. **a** The flow chart depicting the inclusion/exclusion criterion for the predictors. The count of predictors included/excluded at each step is indicated by the number in the parentheses, and the fractions of predictors trained with different training datasets are depicted by the associated pie charts. **b** Precision-recall curves of the top ten predictors in terms of AUPRC on the ALL benchmarking dataset. **c** The statistics of correctly predicted miRNA-disease association pairs among the top 100, top 500, top 1000, and top 5% highly scored predictions on the ALL benchmarking dataset. **d** Precision-recall curves of the top ten predictors in terms of AUPRC on the CAUSAL benchmarking dataset

By manual investigation of the related literature from PubMed and Google Scholar, 90 published miRNA-disease association predictors were collected (Additional file 1: Table S1). However, many of them were not readily available for the benchmarking test. As summarized in Fig. 1a, 3 predictors did not provide available source code or prediction scores, 43 predictors provided partial prediction results that covered only a few diseases or miRNAs, and 8 predictors provided source code but the code failed to run. Finally, 36 predictors, including 16 predictors providing source code and 20 predictors supplying all of their prediction scores, were included in the benchmarking test. Notably, although there were predictors considering datasets other than HMDD v2.0 as their training set, none of these methods met the availability criterion for inclusion (Fig. 1a). Therefore, all of the 36 predictors included in this benchmarking test were trained on the HMDD v2.0 dataset, making them homogeneous but also more comparable in terms of the training dataset.

Our primary independent benchmarking dataset is consisted of all novel miRNA-disease associations in HMDD v3.1 that were not covered by HMDD v2.0. Besides, for reasonable assessment, we performed disease name mapping between HMDD v2.0 and v3.1 and only retained association data with consistent disease names and miRNA names. This dataset, which is referred as the “ALL benchmarking dataset” hereafter, has also been made publicly available at http://www.cuilab.cn/static/hmdd3/data/benchmark2019.txt. We then compared the performance of the 36 readily available predictors on this ALL benchmarking dataset. We noted the prominent imbalanced positive-to-negative ratio of the benchmarking dataset, which resulted from the fact that the number of known miRNA-disease associations, is much smaller than that of possible miRNA-disease combinations. Therefore, rigorous precision-recall curve analysis was adopted to assess the overall performance of these predictors. The top 10 predictors in terms of areas under the precision-recall curve (AUPRC) are shown in Fig. 1b, and the AUPRC results of all predictors are also available (Additional file 1: Table S2). All of the top 10 predictors achieved AUPRC higher than 0.2, suggesting their overall capability for the prediction of miRNA-disease associations. Especially, the first-ranked MCLPMDA (AUPRC = 0.311), the second-ranked LFEMDA (AUPRC = 0.301), and the third-ranked LPLNS (AUPRC = 0.286) exhibited at least 0.05 AUPRC superiority than other methods, highlighting their promising accuracy. MCLPMDA constructed a new miRNA similarity matrix as well as a disease similarity matrix on the basis of matrix completion algorithm before conducting label propagation algorithm in both miRNA space and disease space [15], and this procedure may be helpful to enhance the sensitivity of the algorithm by complementing the unseen miRNA/disease similarity space. LFEMDA designed a new algorithm to obtain the functional similarity than simply using conventional MISIM similarity metrics, and our results suggest the effectiveness of this new miRNA similarity calculation method [23]. Conceptually similar to MCLPMDA, LPLNS also tried to complement the unexplored miRNA-disease association space to improve the performance, but with a distinct weighted nearest neighborhood algorithm [16]. In order to further investigate the performance of predictors that exploited diverged computational frameworks, we classified the 36 predictors into three categories according to the criteria proposed by a recent review [14], i.e., the score function category, the complex network algorithm category, and the machine learning category. The per category comparison results are summarized in Additional file 1: Table S3, where SNMFMDA [51] achieved the highest AUPRC (0.192) in the score function category; MCLPMDA [15] performed best (AUPRC = 0.311) in the complex network algorithm category, and LFEMDA [23] had the superior performance (AUPRC = 0.301) than other predictors in the machine learning category. Together, the better overall performance of these predictors indicates that both a reasonable miRNA similarity metric and effective algorithm for exploring the unseen miRNA-disease associations are important to the performance improvement. On the other hand, the AUPRC is not suitable for assessing the predictor accuracy at specific thresholds. To this end, we further investigated the proportions of correctly predicted miRNA-disease pairs among the top 100, top 500, top 1000, and top 5% highly scored predictions based on the ALL benchmarking dataset. The results are summarized in Fig. 1c, where only MCLPMDA keeps the best ranking at each threshold. Interestingly, the ranks of BLHARMDA [25] and PBMDA [48] significantly ascend to top 5 when investigating their top 100 and top 500 prediction results, indicating their advantages when predicting very high confidence miRNA-disease associations. BNPMDA [19] and HAMDA [29] rank top 3 when considering their top 5% prediction results, suggesting their accuracy in predicting moderately high confidence miRNA-disease associations. Thus, the users may wish to select particular prediction tools based on the number of outputted miRNA-disease association candidates that can be accepted.

While the above results have illustrated the overall performance of the predictors, the prediction accuracy would vary from disease to disease. To preliminarily check the consistency of the evaluation results between different diseases, nine common diseases (melanoma, prostate neoplasms, breast neoplasms, lung neoplasms, gastric neoplasms, ovarian neoplasms, hypertension, type 2 diabetes mellitus, and heart failure) were selected as the typical cases for further evaluation. ROC (receiver operating characteristic) curves were plotted for the top five predictors for each disease (Additional file 2: Figure S1). According to these evaluation results, the predictors showing the best overall prediction performance (i.e., MCLPMDA, LFEMDA, and LPLNS) would still rank in the top five for seven out of the nine common diseases, suggesting their consistency of accuracy. These methods could also rank best for particular diseases. For example, MCLPMDA still achieved the best AUROC (area under ROC curve) in the evaluation for melanoma and ovarian neoplasms, while LFEMDA achieved the best AUROC in the evaluation for breast neoplasms, lung neoplasms, and heart failure. Besides, the performance of NSEMDA [54] is also noticeable as it ranked in top five for five out of the nine common diseases, indicating its advantages in predicting common diseases with extensive miRNA-disease association annotations. Finally, certain predictors would show superior accuracy for one particular disease, such as RFMDA (best for prostate neoplasms) [32], PRMDA (best for gastric neoplasms) [49], BNPMDA (best for hypertension) [19], and MCMDA (best for type 2 diabetes mellitus) [40]. Therefore, these predictors would be the better choices when analyzing the corresponding diseases.

One noticeable issue of the previous HMDD database is that it included all kinds of miRNA-disease associations from literature, but some of them were only supported by weak experimental evidence. For example, a considerable fraction of miRNA-disease associations was derived from the differentially expressed miRNAs in the transcriptome assays which compared the miRNA expression profiles between disease and normal samples, but such simple miRNA differential expression could not support the causal relationship between miRNA and disease. To address this issue, in the past few months, we have performed systematic re-evaluation of the experimental evidence for HMDD v3.1 data to label the potential disease causal miRNAs. As the result, the last version of HMDD (v3.2) provides a new dataset of disease causal miRNAs, which enables us to assemble a CAUSAL benchmarking dataset, a subset of the ALL benchmarking dataset that considers only the causal miRNA-disease associations as the positive testing samples. Intuitively, this CAUSAL benchmarking dataset is much more challenging, since current prediction methods did not aim to distinguish causal miRNA-disease association. As the result, the prediction performance of all predictors is systematically and significantly reduced on the CAUSAL benchmarking dataset (Additional file 1: Table S4). Nevertheless, the ranks of top predictors were largely consistent between the results from the ALL benchmarking dataset (Fig. 1b) and those from the CAUSAL benchmarking dataset (Fig. 1d), among which the ranks of top three predictors have not changed, including the first-ranked MCLPMDA (AUPRC = 0.120), the second-ranked LFEMDA (AUPRC = 0.113), and the third-ranked LPLNS (AUPRC = 0.093). Moreover, nine out of the top ten predictors (MCLPMDA, LFEMDA, LPLNS, SACMDA, ICFMDA, HLPMDA, SNMDA, LLCMDA, and BLHARMDA) were shared between the results on two datasets. As for the per category comparison, MCLPMDA (AUPRC = 0.120) and LFEMDA (AUPRC = 0.113) kept the best rank in the complex network algorithm category and the machine learning category, respectively (Additional file 1: Table S5). In the score function category, the previously second-ranked predictor NARRMDA [44] (AUPRC = 0.063) achieved the highest AUPRC in the score function category, slightly outperforming the previous best-performed predictor SNMFMDA (AUPRC = 0.060).

In all, the consistency of top ranked predictors between the results from the two benchmarking datasets suggests the robustness of these predictors. On the other hand, we also recorded the computational resource and running time required for the methods that are available as the source code to run (Additional file 1: Table S6). Clearly, all of these methods could accomplish the prediction task within 5 min using computational resource affordable by laptops. But the methods adopt a variety of programming languages in their source code, and therefore, a user-friendly interface would be very helpful for non-specialists to implement these methods for their own purpose. As a preliminary effort to increase the accessibility of the prediction algorithms to non-specialists, we have incorporated the prediction results of the nine shared top predictors into the HMDD database (http://www.cuilab.cn/hmdd). The users can either retrieve the results for a particular disease or miRNA from the Browse page of the database, or download the prediction results as a single Excel file (http://www.cuilab.cn/static/hmdd3/data/prediction_combined.xlsx).

While HMDD 3.1 is the largest literature-curated database for miRNA-disease associations to date (at least threefold more records than literature-curated databases according to recent statistics [8]), there are also few databases that infer potential miRNA-disease associations from high-throughput experimental datasets, among which dbDEMC, a database that focuses on the differentially expressed miRNAs in human cancers, is of the highest size [11]. To assess whether the 36 predictors trained with HMDD v2.0 data could also perform well on the heterogeneous dbDEMC dataset, we also test the predictors on the dbDEMC records that were not covered by HMDD v2.0. And the AUPRC results of the top 10 predictors and all predictors are shown in Additional file 1: Table S7 and Additional file 2: Figure S2. Generally, the top 10 predictors achieved an impressive performance on this heterogeneous dataset with an AUPRC over 0.63, where eight of them, including LLCMDA, SNMDA, MCLPMDA, BNPMDA, LPLNS, HLPMDA, ICFMDA, and SACMDA, were also top-ranked on the ALL benchmarking dataset derived from HMDD v3.1, indicating their robustness in predicting cancer-related miRNAs. There are also predictors showing exceptional performance on the dbDEMC dataset. For example, HAMDA [29] and HGIMDA [35] ascended to the top list on the dbDEMC dataset. Nevertheless, cautious interpretation of the dbDEMC results is also required, especially when extending to diseases other than cancer. First, the dbDEMC dataset is composed of the miRNA-disease associations with the weak, differential expression-based evidence, and therefore is not designed to distinguish disease causal miRNAs. Moreover, cancer is the most well-annotated disease in terms of associated miRNAs, and predictors showing an outstanding performance on cancer dataset like HAMDA [29] would be biased to well-studied diseases or miRNAs, a prevalent issue that is further analyzed in the third subsection.

### Iterative integration of predictors could further improve the prediction performance

**Fig. 2:**
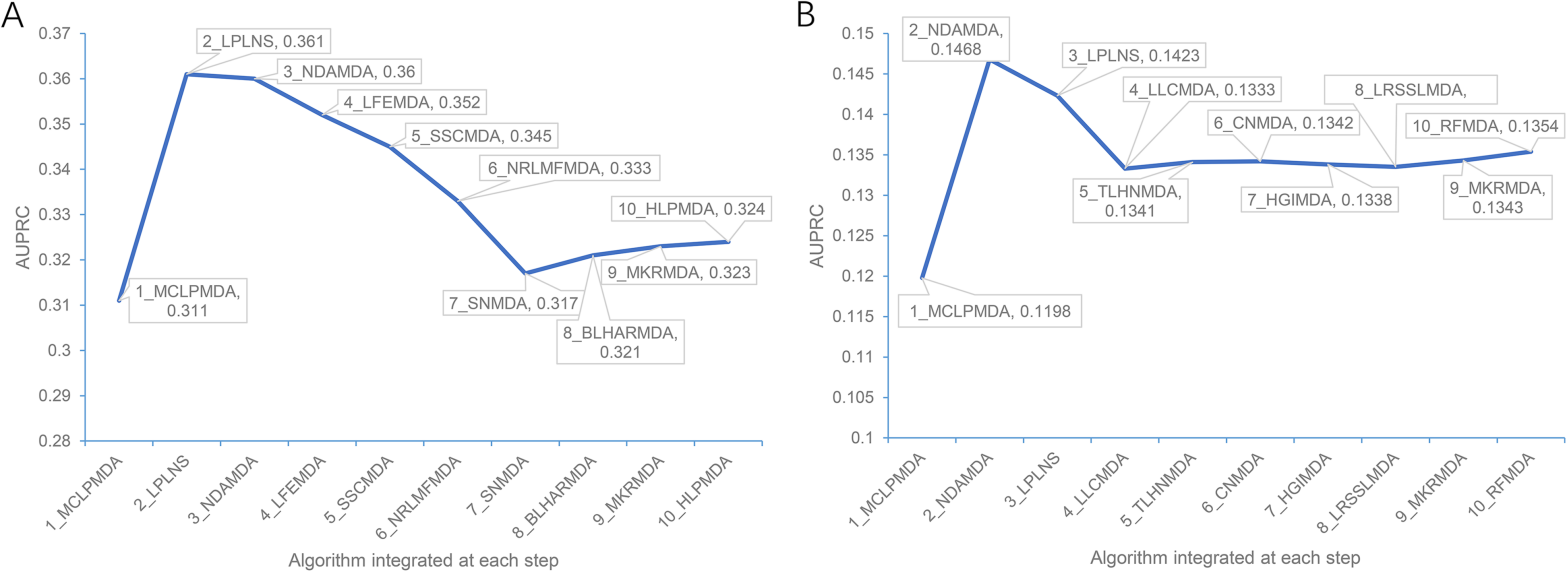
AUPRC improvement with iterative integration of different predictors. The combined predictors using the max-min prediction score normalization approach were tested on the ALL and the CAUSAL benchmarking datasets, respectively. The predictor integrated at each round of iteration and the AUPRC of the combined predictor are indicated on the line chart. **a** The AUPRC results of the combined predictors on the ALL benchmarking dataset. **b** The AUPRC results of the combined predictors on the CAUSAL benchmarking dataset

Notably, the prediction methods have employed various computational approaches and distinct features describing miRNA and/or disease similarity. Therefore, it is likely that some of them are complementary to each other, and integration of such methods could achieve an even better performance. To check this possibility, we first scaled the prediction scores of each predictor to the 0–1 interval by using the max-min normalization approach, and then iteratively integrated their prediction scores with a preliminarily optimized weight of each predictor (see Material and Methods for details). We first performed the predictor combination process on the ALL benchmarking dataset. The iteration started from MCLPMDA predictor which has the highest observed AUPRC (Additional file 1: Table S2). Integration with LPLNS [16] resulted in the best AUPRC improvement to 0.361 at the second round of iteration. Both MCLPMDA and LPLNS were based on label propagation algorithm, but MCLPMDA further incorporate the matrix completion algorithm while LPLNS used linear neighborhood similarity in the network [15, 16]. Further integration of NDAMDA [34], another complex network algorithm exploiting additional network distance features, could also result in a similar AUPRC of 0.360 (Fig. 2a). The variation in their algorithm framework, especially in the description of complex network features, might be helpful to establish the mutual complementarity between these three methods. After the third round of iteration, the AUPRC began to drop until the eighth iteration (Fig. 2a). Nevertheless, the performance of the combined predictors at either round of iteration outperformed the best single predictor MCLPMDA, indicating that the predictor combination is indeed helpful to the performance improvement. The same iterative predictor combination process was also performed on the CAUSAL benchmarking dataset, and a similar trend of AUPRC was observed (Fig. 2b). Starting from the best-performed predictor MCLPMDA (Additional file 1: Table S4), sequential integration with NDAMDA and LPLNS resulted in the combined predictors showing the best two AUPRCs (0.147 and 0.142, respectively). Together, the above results demonstrate the possibility and effectiveness of method combination. Besides, we also noted that the consensus combination of the first three predictors between two predictor combination processes (MCLPMDA + LPLNS + NDAMDA). Therefore, to facilitate the community, similar to the nine shared top-ranked predictors selected in the previous subsection, we also made the prediction score of these three predictors and their combination available on the HMDD database (http://www.cuilab.cn/hmdd).

### Assessing the potential bias from the overrepresented miRNAs and diseases in the current dataset

**Fig. 3:**
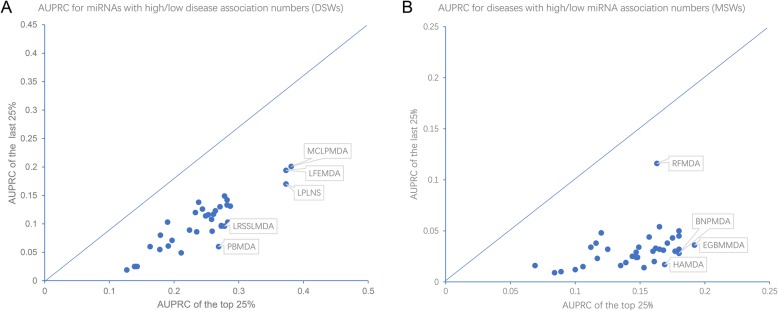
The stratified comparison of predictor performance in terms of DSW and MSW. **a** Dot plots where the AUPRCs of the well-annotated miRNAs (with the top 25% DSW scores) are plotted against AUPRCs of the less-annotated miRNAs (with the last 25% DSW scores). **b** Dot plots where the AUPRCs of the well-annotated diseases (with the top 25% MSW scores) are plotted against AUPRCs of the less-annotated diseases (with the last 25% DSW scores)

The miRNA-disease association pairs are not evenly distributed amid the possible miRNA-disease combinations. According to the published statistics of HMDD v2.0 [24] and that of more recent HMDD v3.0 [8], a few miRNAs like hsa-miR-21 show extraordinary amounts of associated diseases, while several prevalent cancer types dominate the top-ranked list of diseases with the highest numbers of associated miRNAs. Such overrepresentation of specific miRNAs or diseases would predispose bias in the prediction models, where well-annotated miRNAs or diseases tend to have much better prediction accuracy. To check this possibility, we first stratified the prediction results of different miRNAs based on their disease spectrum width (DSW). Higher DSW scores indicate wider disease associations of miRNAs [8]. Figure 3a compares each predictor’s performance between the well-annotated miRNAs (with the top 25% DSW) and the less-annotated miRNAs (with the last 25% DSW). As intuitively expected, all predictors show much better performance for well-annotated miRNAs than less-annotated miRNAs, with the average AUPRC of the former ones as about twofold as large as that of the latter ones. Nevertheless, the AUPRC differences between two DSW groups are largely comparable among the predictors, indicating that there is no particular computational framework susceptible to the bias from the overrepresentation of well-annotated miRNAs in the dataset. Only three prediction methods, including PBMDA, LRSSLMDA, and LPLNS, show slightly higher preference toward high DSW miRNAs. Interestingly, we also noted MCLPMDA, the top-ranked predictor in overall AUPRC assessment (Fig. 1), show the best AUPRC for both DSW groups. Therefore, developers may consider to integrate this computational approach or its conceptual idea to build a more robust predictor that could accurately predict less-annotated miRNAs.

A similar measurement named miRNA spectrum width (MSW) [8] could be used to stratify the well- and less-annotated diseases in terms of their miRNA associations. Accordingly, we also compared the AUPRC between the well-annotated diseases (with the top 25% MSW) and the less-annotated diseases (with the last 25% MSW). As intuitively observed in Fig. 3b, the situation is much severe for the MSW stratification than that for the DSW stratification. All predictors show much worse performance for less-annotated diseases than well-annotated diseases, and on average, the fold change of AUPRC between the two groups could reach four- to fivefold. For the predictors showing the largest performance differences between two groups (HAMDA, EGBMMDA, and BNPMDA), the fold changes could be further raised over fivefold, until tenfold. These results highlight the noteworthy problem that most of current prediction methods are susceptible to the overrepresented diseases in the dataset and therefore tend to be significantly biased toward well-annotated diseases. Unfortunately, by surveying the related references, we also noted that the developers tended to use data for well-annotated diseases like cancers to exemplify the effectiveness of their predictors. For example, dbDEMC, a database collecting differentially expressed miRNAs in cancers [10, 11], is often introduced as the additional validation data for the predictors. As clearly shown by the above analysis results, predictor performance for the well-annotated diseases like cancers does not constitute a good representation of the performance for the less-annotated diseases. This is also demonstrated by the diverged performance assessment results between the HMDD dataset and dbDEMC database for some predictors like HAMDA (Tables S2 and S7). Therefore, special focus on the less-annotated diseases is necessary to further improve the robustness of the predictors. On the other hand, one predictor, RFMDA [32], shows comparable performance across both the high MSW group (AUPRC = 0.163) and low MSW group (AUPRC = 0.116), indicating it is much less biased toward well-annotated diseases. Further development of predictors may consider including its feature vector schema to improve the predictor’s performance on less-annotated diseases.

### A preliminary comparison between MISIM 1.0 and MISIM 2.0 miRNA functional similarity matrixes

**Fig. 4:**
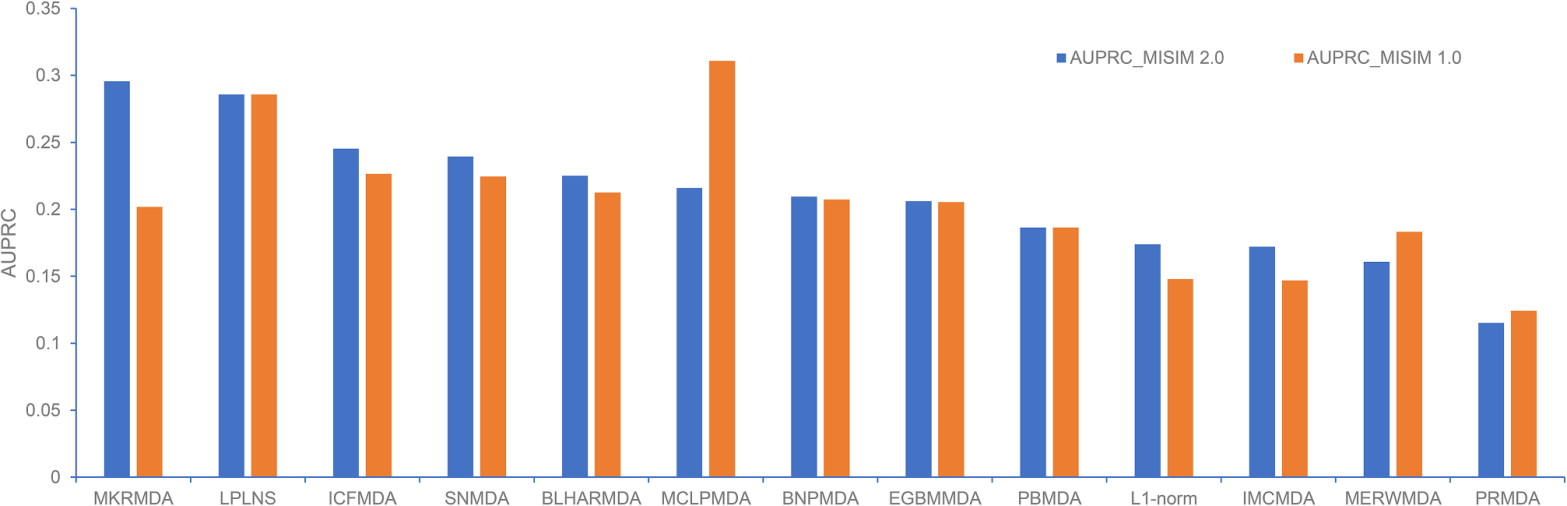
The comparison of the prediction performance using MISIM 2.0 or MISIM 1.0 miRNA similarity matrix

In line with the guilt-by-association principle to infer biological functions, functionally similar miRNAs should tend to co-regulate the phenotypically similar diseases. Therefore, most of the prediction methods have employed the functional similarity between miRNAs as one of the core component in their algorithms, among which the MISIM (or more specifically MISIM v1.0) miRNA functional similarity matrix has been most widely adopted [52]. Indeed, 13 out of the 16 predictors available as a source code used MISIM v1.0 as (one of) their primary miRNA similarity metric(s). Recently, MISIM v2.0 has been released based on the novel data from the HMDD v3.0 database [53]. Therefore, it is interesting to investigate if the predictors would benefit from this more updated miRNA similarity matrix. To this end, we replaced the MISIM v1.0 similarity matrix with MISIM v2.0 and re-ran the programs to obtain new prediction scores for the 13 models. Then the performances based on two similarity matrixes were compared on the same benchmarking dataset described above. The testing results are summarized in Fig. 4. Most methods except MCLPMDA, MERWMDA, and PRMDA exhibit performance improvement to different extents when using MISIM v2.0, where MKRMDA benefits the most, with a 0.085 augmentation of AUPRC. On the other hand, MCLPMDA shows a noticeable AUPRC decrease (0.095) with the MISIM v2.0. MCLPMDA implemented the matrix completion algorithm specifically designed on the previous miRNA and disease similarity matrixes, and it seems necessary to re-design the matrix completion algorithm based on the new MISIM v2.0 data to efficiently exploit this novel miRNA functional similarity matrix. In all, the new miRNA functional similarity matrix MISIM v2.0 would be helpful to improve the prediction performance, but careful algorithm design is required to deal with the differences between MISIM v1.0 and v2.0, in order to make full use of this new similarity matrix.

### Prioritizing disease causal miRNAs from general disease-associated miRNAs is still a challenging task

**Fig. 5:**
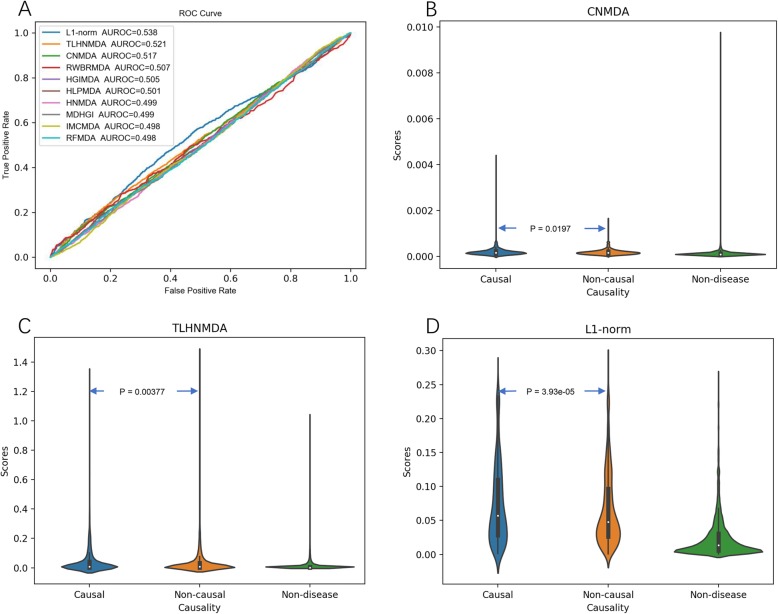
The prediction performance for prioritizing disease causal miRNAs. **a** The ROC curves illustrating the performance in distinguishing causal miRNA-disease associations (as the positive samples) from the non-causal miRNA-disease associations (as the negative samples); only the top ten predictors in terms of AUROC are shown. **b**–**d** The violin plots for three predictors that show significant higher prediction scores (via Wilcoxon test) for causal miRNA-disease associations than non-causal miRNA-disease associations

MiRNAs have different roles in diseases. Some causal miRNAs could directly participate in the mechanisms of the diseases, while others only show non-causal associations with the diseases (e.g., simply exhibiting differential expression without furtherer mechanism evidence). Therefore, identifying the potential disease causal miRNAs is crucial for understanding the underlying mechanism of diseases. Until recently, there is no comprehensive annotation dataset about the disease causal miRNAs. Therefore, current miRNA-disease association prediction methods do not aim at distinguishing disease causal miRNAs. To address this issue, in the latest HMDD v3.2 version, although no additional miRNA-disease associations were included in comparison with HMDD v3.1, a manual curated causal miRNA-disease association dataset was made available. This new dataset gives us an opportunity to test whether the current predictors, which have been designed to predict general miRNA-disease associations, could also prioritize the disease causal miRNAs. For this purpose, we divided all miRNA-disease pairs in the benchmarking dataset into three groups, i.e., “causal,” “non-causal,” and “non-disease.” In the first subsection above, we have tested the ability of predictors to distinguish the “causal” pairs from the “non-disease” pairs by using the CAUSAL benchmarking dataset (Fig. 1d). Here, we went a step further to evaluate the predictors for discriminating the “causal” (as the positive samples) and “non-causal” (as the negative samples) pairs by their AUROCs. This is a very challenging task since either “causal” or “non-causal” miRNA-disease associations were considered as the positive samples when training the miRNA-disease association predictors and no further stratification of the positive samples according to the disease causality has been considered. The evaluation results are summarized in Fig. 5a and Additional file 1: Table S8. Unfortunately, none of the predictors achieve satisfactory performance in distinguishing causal and non-causal miRNAs, where the best AUROC is limited to 0.538. Therefore, we took a relaxed approach by comparing the prediction scores between causal and non-causal miRNAs using the Wilcoxon statistical test. Among the 36 predictors, only three methods show significant higher prediction scores for causal miRNAs than non-causal ones, including L1-norm (*P* value = 3.93e–05), CNMDA (*P* value = 0.0197), and TLHNMDA (*P* value = 0.00377), indicating a weak potential for distinguishing the causal miRNAs of these methods. Nevertheless, the overall performance of these predictors for general miRNA-disease associations are not very impressive (Additional file 1: Table S8), suggesting that additional biological features are required for the distinction between disease causal miRNAs and generally associated miRNAs. Therefore, newly designed computational approaches based on the new disease causal miRNA dataset are highly warranted to efficiently identify causal miRNA-disease associations. With the increasing research interests on the mechanisms of miRNAs involved in diseases, causal miRNA prediction is very likely to become an emerging important direction for the related bioinformatics studies in the near future.

## Conclusion

Rapidly increasing evidence has demonstrated that miRNAs are involved in the onset and development of a wide spectrum of human diseases, which has further propelled the emergence of miRNA-disease association prediction being an active direction in the field of bioinformatics. Here, we systematically evaluated 36 predictors, which were established using various machine learning algorithms and network analysis methods, on an independent benchmarking dataset consisting of more than 8000 novel miRNA-disease associations. Here, by outlining the above benchmarking results, several points that would be helpful to the users and developers of the predictors could be highlighted. As for the users of miRNA-disease prediction tools, (1) many prediction methods do not have publish their pre-calculated prediction results or source codes, and we have summarized the 36 readily available tools (Additional file 1: Table S1) for further consideration. (2) All of the high-ranked predictors exhibited acceptable overall performance in the benchmarking test, with the top 13 predictors reaching AUPRC > 0.2, and the MCLPMDA, LFEMDA, and LPLNS achieved the best overall performance (Fig. 1). (3) Users should be cautious of the potential bias toward the overrepresented diseases. That is to say, current predictors tend to show a much better performance in predicting miRNAs of well-studied diseases like cancers compared to their performance in predicting less-studied diseases. For now, RFMDA is one solution to predict miRNA-disease associations for less-studied diseases (Fig. 3), but combination of prediction results with other experimental data should be encouraged. (4) Current predictors do not tend to prioritized disease causal miRNAs; therefore, the prediction scores cannot be considered as a primary reference for screening target miRNAs for further disease mechanism studies. As for the developers of miRNA-disease prediction tools, (1) current predictors adopted different programing languages in their source code (Additional file 1: Table S6), and therefore, a user-friendly interface or a webserver is encouraged to facilitate the biomedical researchers who are not familiar with the prediction pipelines. One example is RWRMTN [55], which provides a querying interface of its top prediction results as a Cytoscape plugin. (2) The best performed algorithms (Fig. 1) like MCLPMDA often take various approaches to explore the unseen miRNA-disease associations, which may be helpful to the robust performance on the independent dataset. (3) Integrating different predictors as a meta-predictor (Fig. 2) or updating the miRNA functional similarity matrix (Fig. 4) would also improve the predictors’ performance. (4) The developers should be aware of the bias toward well-annotated diseases (Fig. 3), and the predictor performance among the diseases with few known miRNA associations should be intentionally checked to reduce such bias. (5) Current predictors do not design for screening disease causal miRNAs (Fig. 5), and novel computational approaches are highly warranted to effectively prioritize the disease causal miRNAs from general miRNA-disease associations, perhaps based on the latest disease causality annotation from HMDD v3.2. On the other hand, current benchmark test also has its own limitations. First, although HMDD v3.1 could constitute a sizable miRNA-disease association dataset for a benchmarking analysis, its coverage is still not fully satisfactory compared to the possible miRNA-disease combinations. Therefore, continuous benchmarking of the predictors with newly discovered miRNA-disease associations is necessary. Second, a considerable number of prediction methods were not included because of their limited availability. A larger-scale benchmarking test, when these predictors become available, will clearly benefit the potential users to find more competent tools for analyzing the miRNA-disease associations. Together, we hope our benchmarking analysis would serve as a helpful reference for biomedical researchers to choose appropriate predictors as well as a hint about the future directions for predictor improvements.

## Materials and methods

### Inclusion and exclusion criteria of the prediction methods

By querying PubMed and Google Scholar with the keywords “miRNA-disease + prediction,” 118 related references were obtained. After surveying on the literature full text or software homepage, 90 predictors were curated as the candidates for benchmarking analysis (Additional file 1: Table S1). Unfortunately, however, we found more than half of these predictors did not have a readily available tool or prediction score for further assessment, and only 37 are readily available either as source code, standalone software, or pre-calculated prediction scores. During further assessment, one tool was excluded because it produced few confidence levels rather than exact prediction scores [7]. We also noted that although ~ 15% of the candidate predictors used training datasets other than HMDD v2.0, as for the readily available tools, all of them were trained only with HMDD v2.0 dataset (Fig. 1a). As the result, 36 available prediction methods trained with HMDD v2.0 dataset were finally included in this benchmarking analysis.

### Benchmarking test and performance statistics

The newly curated experimental miRNA-disease associations from the HMDD v3.1 database (http://www.cuilab.cn/static/hmdd3/data/alldata.txt) that were not covered by HMDD v2.0 (http://www.cuilab.cn/static/hmdd3/data/hmdd2.zip) were obtained as the primary benchmarking samples. Because the disease nomenclature has changed from “MeSH” in HMDD v2.0 to “Disease Ontology + MeSH” in HMDD v3.1, the disease name mapping from HMDD v3.1 back to HMDD v2.0 was performed on all benchmarking samples to avoid false negative artifacts resulting from the inconsistency of disease names. The newly reported diseases or miRNAs in HMDD v3.1 were not included in the benchmarking test. As the result, the ALL benchmarking dataset covers 7178 novel miRNA-disease associations, which can be downloaded at http://www.cuilab.cn/static/hmdd3/data/benchmark2019.txt, and the disease name mapping file from HMDD v3.1 to HMDD v2.0 was also made available at http://www.cuilab.cn/static/hmdd3/data/disease_mapping2019.txt. Besides, based on the disease causality labels of miRNA-disease association in HMDD v3.2 (http://www.cuilab.cn/hmdd#fragment-8), the CAUSAL benchmarking dataset was further extracted by limiting the causal miRNA-disease associations as the positive samples. The CAUSAL benchmarking dataset covers 2339 novel miRNA-disease associations, which can be downloaded at http://www.cuilab.cn/static/hmdd3/data/benchmark2019_causal.txt. Finally, we also compiled a testing dataset from dbDEMC, a database collecting differentially expressed miRNAs in various cancer types (36) [11]. The dbDEMC dataset covers 7616 potential miRNA-disease associations that were not covered by HMDD v2.0, and this dataset can also be downloaded at http://www.cuilab.cn/static/hmdd3/data/benchmark2019_dbDEMC.txt.

The prediction scores on the benchmarking samples were either fetched from the pre-calculated prediction results or obtained by re-running the source code on our computer (CPU: Intel® Core™ i7-7700 CPU @ 3.6 Hz, 8 cores; Memory: 8 GB; see Additional file 1: Table S6 also for the required computational resource). Note that the prediction scores obtained by either approach are largely consisted of a D × M matrix where D and M are the numbers of HMDD v2.0 diseases and miRNAs that could be mapped to the benchmarking dataset, respectively. Therefore, the size of the prediction score matrixes for different prediction tools are roughly equivalent, no matter how the prediction scores were pre-calculated or derived from the re-running of the program. Based on the prediction scores, we plotted the precision-recall curve for each prediction method and calculated AUPRC as the primary performance evaluation metric by using the *sklearn* package in Python. Besides, we also ranked the prediction scores for each predictor to investigate the proportions of correctly predicted miRNA-disease pairs among the top 100, top 500, top 1000, and top 5% highly scored predictions, respectively.

### Iterative integration of the prediction methods

The prediction scores from each predicator were firstly normalized to a 0–1 interval via the max-min normalization approach to avoid the scaling issue when performing further combination:

$$ {x}^{\ast }=\frac{x-{x}_{\mathrm{min}}}{x_{\mathrm{max}}-{x}_{\mathrm{min}}} $$

where *x* means the score of one miRNA-disease pair to be normalized, *x*_min_ and *x*_max_ indicate the minimum and maximum scores among all prediction scores of one predictor. We also compared the results from the max-min normalization approach with those from the other two popular approaches (i.e., *Z*-score normalization and Sigmoid normalization) as well as those without any normalization, and found that the four approaches showed similar results with respect to the AUPRC of the combined predictors (Additional file 2: Figure S3). Then we started with the predictor showing the highest AUPRC and iteratively combined the prediction scores of other predictors. For each round of iteration, we added one predictor that could improve the AUPRC to the most. The predictors were combined based on the weighted summing of their normalized prediction scores, where the weight of each single predictor was optimized (taking 0.05 as the step size) and renewed for every possible predictor combination at each round of iteration. We ran 10 rounds of iterations, but the performance began to drop from the third or fourth iteration (Fig. 2), indicating the optimized predictor combination could be selected within few rounds of iteration.

### Stratified analysis of the prediction scores based on MSW and DSW

MicroRNA spectrum width (MSW) and disease spectrum width (DSW) are a pair of metrics that can roughly assess the overrepresentation of certain diseases and miRNAs in current miRNA-disease association data, where well-annotated diseases and miRNAs can be indicated by high MSW and high DSW scores, respectively. Based on the latest HMDD v3.1 data, we re-calculated the DSW and MSW scores following the previously described method [8]. We then stratified the prediction scores for the miRNAs in the top 25% DSW interval and those in the last 25% DSW interval, and compare AUPRC among these two groups. The same comparison was also performed based on the MSW stratification.

### MISIM 2.0 vs MISIM 1.0 comparison

For the 13 predictors (as listed in Fig. 4) which provided source codes and adopted MISIM v1.0 as their miRNA functional similarity matrix, we tried to replace their similarity matrix with the MISIM v2.0 and re-ran the codes to check the change of AUPRC on the benchmarking set. The MISIM v2.0 miRNA similarity matrix was obtained from the website (using the one not including up-/downregulation, i.e., http://www.lirmed.com/misim/similarity.zip). Note that all new miRNAs in MISIM v2.0 that were not covered by the previous MISIM v1.0 matrix were removed before the subsequent calculations.

### Predicting disease causal miRNAs

Based on the disease causality labels of miRNA-disease association in HMDD v3.2 (http://www.cuilab.cn/hmdd#fragment-8), we grouped the miRNA-disease pairs in the benchmarking dataset to “causal” pairs and “non-causal” pairs, respectively. The capability of the predictors to prioritize the disease causal miRNAs was assessed by ROC plot and AUROC values, where the “causal” pairs were assigned as the positive samples and the “non-causal” one was assigned as the negative samples.

## Supplementary information

Additional file 1: Supplementary tables. This additional file includes 8 supplementary tables, i.e. **Tables S1-S8**. (DOCX 34 kb)

Additional file 2: Supplementary figures. This additional file includes 3 supplementary figures, i.e. **Figures S1-S3.** (PDF 1430 kb)

**Additional file 3.** Review history. (DOCX 21 kb)

## Data Availability

The authors declare that the data supporting the findings of this study are available within the article, its supplementary information files and the public HMDD database (http://www.cuilab.cn/hmdd/). Besides, the benchmarking datasets and related codes were also available at GitHub [56] and Zenodo [57], respectively.
