# Supplementary material for: Benchmark of computational methods for predicting microRNA-disease associations
Source: Genome Biol. 2019 Oct 8;20:202. doi: 10.1186/s13059-019-1811-3 (PMC6781296; doi:10.1186/s13059-019-1811-3)
Supplement: Supplementary file 2 — Supplementary figures. This additional file includes 3 supplementary figures, i.e. Figures S1-S3. (PDF 1430 kb) [file 13059_2019_1811_MOESM2_ESM.pdf]

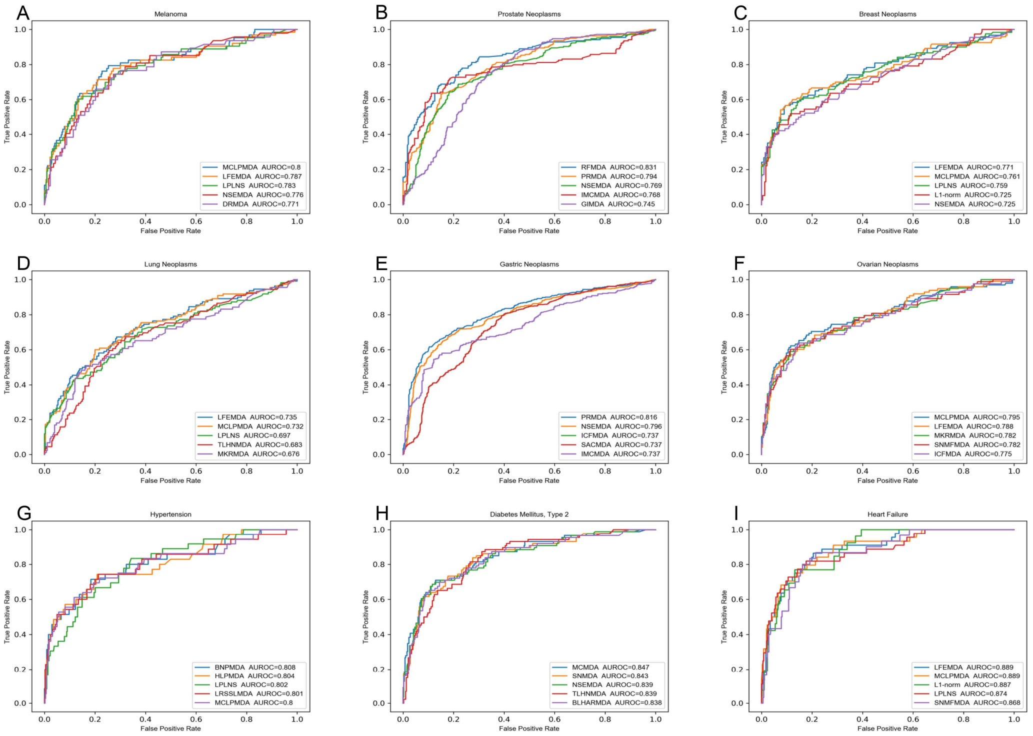

Figure S1. The ROC plots of the top five predictors that performed best in each disease assessment.

(A) The results of melanoma assessment. (B) The results of prostate neoplasms assessment. (C) The results of breast neoplasms assessment. (D) The results of lung neoplasms assessment. (E) The results of gastric neoplasms assessment. (F) The results of ovarian neoplasms assessment. (G) The results of hypertension assessment. (H) The results of Diabetes Mellitus, Type 2 assessment. (I) The results of heart failure assessment.

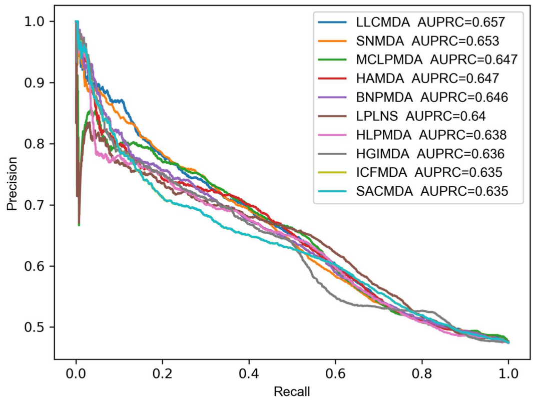

Figure S2. Precision-recall curves of the top ten predictors in terms of AUPRC on the dbDEMC dataset.

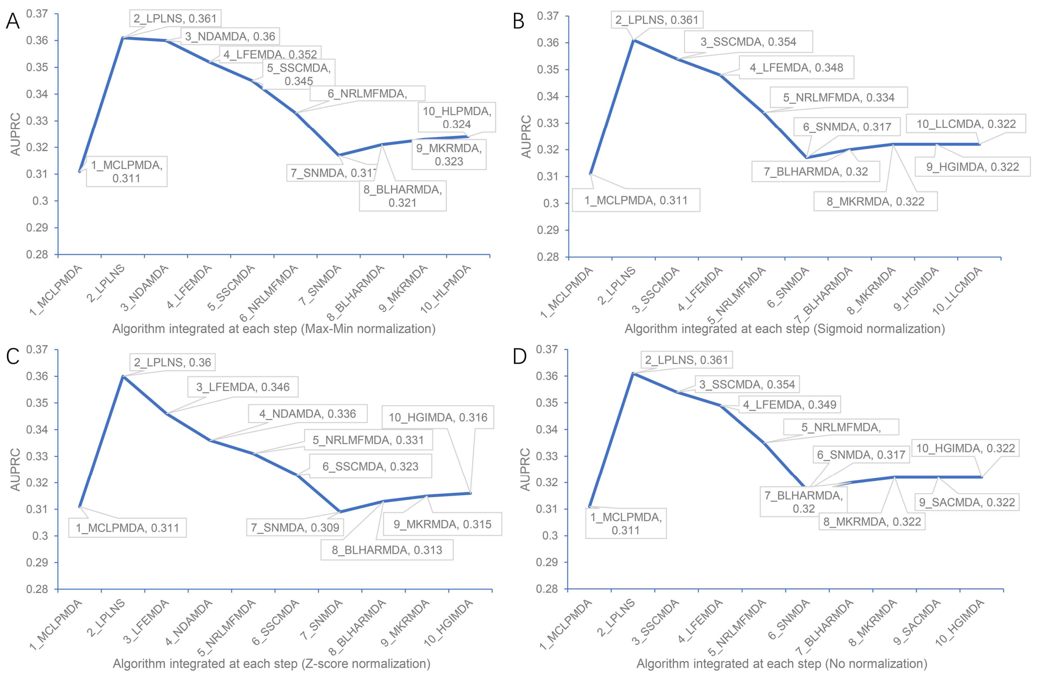

Figure S3. AUPRC improvement with iterative integration of different predictors, using different prediction score normalization approaches.

The combined predictors using different prediction score normalization approaches were tested on the ALL benchmarking dataset. The predictor integrated at each round of iteration and the AUPRC of the result combined predictor are indicated on the line chart. (A) The result using the Max-Min normalization approach. (B) The result using the Sigmoid normalization approach. (C) The result using the Z-score normalization approach. (D) The result without normalization of the prediction scores.
