# Supplementary material for: Benchmark of computational methods for predicting microRNA-disease associations
Source: Genome Biol. 2019 Oct 8;20:202. doi: 10.1186/s13059-019-1811-3 (PMC6781296; doi:10.1186/s13059-019-1811-3)
Supplement: Supplementary file 3 — Additional file 3. Review history. (DOCX 21 kb) [file 13059_2019_1811_MOESM3_ESM.docx]

Review History

**First round of review**

**Reviewer 1**

**Are you able to assess all statistics in the manuscript, including the appropriateness of statistical tests used?**

yes

**Comments to author:**

The authors present a bioinformatics analysis to compare the performance of various public algorithms for predicting miRNA-disease associations. At present, many bioinformatics tools have been published in this field, and thus it would be important to objectively assess the quality of these prediction tools. To this end, the current study is an interesting attempt and could be helpful for researchers to choose the best tools or algorithms for the prediction of miRNAs involving in specific diseases of interest. Overall, this is an interesting comparative analysis based on newly curated miRNA-disease association data as presented in the most updated version (v3.0) of the HMDD database. The authors of this study recently added thousands of new HMDD entries, which represent independent data for tool comparison. The results from the current study would provide valuable guidance for miRNA researchers.  Overall, this is a well presented study. The comparison analysis is quite comprehensive, including 36 bioinformatics tools that are freely accessible to the public. Besides tool comparison, this study also presents some interesting observations, such as severe biases towards well-annotated miRNAs/diseases, which are valuable information to miRNA researchers.

However, there are also concerns with this study that should be addressed before publication.

1) The authors chose to include tools that were trained solely with HMDD data. This selection strategy would significantly limit the scope of the comparison analysis. As all included algorithms were trained using the same dataset, their performance tends to converge. It is important to include tools that were developed using alternative data strategies such as training with non-HMDD data or bioinformatics prediction data. 

2) The authors attempted to combine multiple HMDD-trained tools for performance improvement (Fig. 2). However, the improvement is relatively minor. A larger improvement might be possible if relatively independent tools (e.g. trained with HMDD vs. non-HMDD data) are combined. 

3) It would be very helpful to provide recommendations based on the performance comparison of various tools. Currently, there are numerous tools available, which makes it difficult for non-expert users to select the most appropriate tools for their research. It would be even better if the best performing tools (or a tool assemble) are presented in HMDD as an optional bioinformatics extension of the manually curated literature data.

4) Replace "researches" with "studies". The figure resolution is poor. Consider changing the image format to line art vector.

**Reviewer 2**

**Are you able to assess all statistics in the manuscript, including the appropriateness of statistical tests used?**

There is no statistics

**Comments to author:**

In this manuscript, the authors aim to evaluate and compare the performance of 36 microRNA- disease prediction methods using a benchmark dataset downloaded from the HMDD3.0 database. It is meaningful to evaluate the performance of various tools and find the best prediction tools. However, several concerns have to be addressed as shown below.
Major points:
1. the 36 miRNA-disease prediction tools assessed in this manuscript were developed by only several authors. Up to 27 of the 36 tools are developed by Dr. Chen X, who might be better suited to evaluate the performance of these tools, or other scientists look through the latest prediction papers from the Dr. Chen's group to find out which method is the best. 

2. the benchmark dataset from the HMDD3.0 database is not very well for predicting new miRNA-disease associations, because the benchmark dataset includes many unreliable microRNA-disease associations that microRNA is only differentially expressed in disease compared to normal inferred from high-through technologies such as microarray.  It sounds unreasonable to take unreliable data as training data to predict new microRNAs-disease associations.

3. the topic of predicting disease-related microRNAs is not very new, which has been studied for nearly ten years. Although many tools for predicting disease-related microRNAs have been published, few biologists have used these prediction tools to guide their biological experiments for verifying true miRNA-disease associations.

Minor points:
1. it may be much better that the 36 tools are categorized to evaluate the performance of each method in each category.

2. In ‘Benchmarking test and performance statistics’ section, the authors mapped all the disease terms in HMDD v2.0 and HMDD v3.0 to Disease Ontology (DO) and MeSH as benchmark set. The two ontologies have different structures, and parts of the 36 methods depend on the uniform structure of the ontology. It is confusing how to predict the novel disease-miRNA associations for those disease terms occurring only in the MeSH or DO.

3. the link (http://www.cuilab.cn/hmdd3/data/disease_mapping2019.txt) is not accessible.

4. the authors mentioned the prediction scores from each predicator were normalized to 0-1. It is not clear how to normalize the scores? Is it suitable for those classification-based prediction tools?

**Authors Response**

**Point-by-point responses to the reviewers’ comments:**

**Reviewer 1**

The authors present a bioinformatics analysis to compare the performance of various public algorithms for predicting miRNA-disease associations. At present, many bioinformatics tools have been published in this field, and thus it would be important to objectively assess the quality of these prediction tools. To this end, the current study is an interesting attempt and could be helpful for researchers to choose the best tools or algorithms for the prediction of miRNAs involving in specific diseases of interest. Overall, this is an interesting comparative analysis based on newly curated miRNA-disease association data as presented in the most updated version (v3.0) of the HMDD database. The authors of this study recently added thousands of new HMDD entries, which represent independent data for tool comparison. The results from the current study would provide valuable guidance for miRNA researchers. Overall, this is a well presented study. The comparison analysis is quite comprehensive, including 36 bioinformatics tools that are freely accessible to the public. Besides tool comparison, this study also presents some interesting observations, such as severe biases towards well-annotated miRNAs/diseases, which are valuable information to miRNA researchers. However, there are also concerns with this study that should be addressed before publication. 

**Response: Thank you for your positive comments and constructive suggestions.* 

1) The authors chose to include tools that were trained solely with HMDD data. This selection strategy would significantly limit the scope of the comparison analysis. As all included algorithms were trained using the same dataset, their performance tends to converge. It is important to include tools that were developed using alternative data strategies such as training with non-HMDD data or bioinformatics prediction data. 

**Response: Thank you for your insightful comment. According to your suggestion, we have performed a more comprehensive literature re-curation to seek miRNA-disease association methods trained in datasets other than HMDD v2.0, and the curation result is detailed in Supplementary Tables S1 in the revised manuscript. Among the curated 90 methods, there are indeed a few predictors trained with non-HMDD v2.0 data (14 out of 90, including the cases trained with the older HMDD v1.0). Unfortunately, however, none of these predictors provided a publicly available code nor a sizable list of prediction results. The largest available one, RWRMTN, only provided results covering 35 diseases and 740 miRNAs, while many more predictors only provided results for few common cancer types. Therefore, no additional predictor meets the inclusion criterion of this benchmark study. To intuitively clarify this situation, we have included a flow chart to explain the predictor inclusion/exclusion process of this benchmark study (Figure 1A). 

Nevertheless, we acknowledge your concerns related to this point, i.e. predictor combination performance and requirement of heterogeneous dataset. For the former issue, we found that optimizing the weight of each predictor could substantially increase the performance of the combined predictor (please see our response to the next point for details). For the later one, we also tested the predictors on a new, heterogeneous dataset from dbDEMC, a database collecting de-regulated miRNAs in cancers derived from transcriptome datasets rather than literature. The benchmarking result is also described in the revised manuscript (Page 13 and Supplementary Figure S1). Briefly, 8 out of the top 10 predictors on HMDD v3.1 dataset also presented in the top list on the dbDEMC testing dataset (Supplementary Figure S1 vs. Figure 1B), indicating a well agreement between the results from these two heterogeneous datasets.* 

2) The authors attempted to combine multiple HMDD-trained tools for performance improvement (Fig. 2). However, the improvement is relatively minor. A larger improvement might be possible if relatively independent tools (e.g. trained with HMDD vs. non-HMDD data) are combined. 

**Response: Thank you for your constructive suggestion. As clarified in our response to the previous point, there is no additional tools trained with non-HMDD data readily available for inclusion. 

Nevertheless, we also noted that our previous strategy for predictor combination. i.e. using AUPRC as the arbitrary predictor weight, would limit the performance of the combined predictor. Therefore, we re-performed the predictor combination process by optimizing the weight of each predictor in a step-wise manner. The results suggest that the maximum performance could reach an AUPRC of 0.36, a substantial improvement compared to the previous combination (Figure 2A). Moreover, as suggested by Reviewer #2, we also performed a specific benchmarking test on the disease causal miRNAs. Interestingly, we found the top 3 predictors selected during the predictor combination processes tend to converged between these two benchmarking tests (i.e. disease vs. non-disease and causal vs. non-disease, Figure 2). Therefore, in addition to describing the predictor combination results in revised manuscript (Page 14), we also included the prediction results combing these three predictors to our HMDD database (http://www.cuilab.cn/hmdd/).* 

3) It would be very helpful to provide recommendations based on the performance comparison of various tools. Currently, there are numerous tools available, which makes it difficult for non-expert users to select the most appropriate tools for their research. It would be even better if the best performing tools (or a tool assemble) are presented in HMDD as an optional bioinformatics extension of the manually curated literature data. 

**Response: We fully agree with this comment. In the revised manuscript, we have performed two benchmarking tests on HMDD v3.1 dataset, i.e. disease vs. non-disease and causal vs. non-disease. Thus, the prediction results of the selected predictors from both benchmarking tests were incorporated into the HMDD database. More specifically, we noted that the top 3 predictors during prediction combination process tend to converge between these two tests. Therefore, the prediction results of the combined predictor as well as those of the three individual predictors have been incorporated to the HMDD database. Moreover, 9 out of the top 10 predictors were also shared between two tests, and therefore the prediction results of these 9 shared top predictors were also incorporated into the HMDD database. These prediction results can either be queried by the Browse tool of HMDD database, or batch downloaded from the Download page of the database (http://www.cuilab.cn/static/hmdd3/data/prediction_combined.xlsx ).* 

4) Replace "researches" with "studies". The figure resolution is poor. Consider changing the image format to line art vector. 

**Response: We have done the replacement. As for the figure resolution issue, we have replotted the figures to improve the resolution, but this issue seems to actually result from the figure compression process of the online manuscript system when building the PDF file for review. Thus, you may wish to view the high-resolution images by clicking the corresponding link to each figure file (at the upper-right corner of each figure).* 


**Reviewer 2**
In this manuscript, the authors aim to evaluate and compare the performance of 36 microRNA- disease prediction methods using a benchmark dataset downloaded from the HMDD3.0 database. It is meaningful to evaluate the performance of various tools and find the best prediction tools. However, several concerns have to be addressed as shown below. 

**Response: Thank you for your positive comments and constructive suggestions.* 

Major points: 
1. the 36 miRNA-disease prediction tools assessed in this manuscript were developed by only several authors. Up to 27 of the 36 tools are developed by Dr. Chen X, who might be better suited to evaluate the performance of these tools, or other scientists look through the latest prediction papers from the Dr. Chen's group to find out which method is the best. 

**Response: Thank you for the comment and we indeed noted the important contribution by Dr. Chen to this field. Nevertheless, it turns out that looking through the latest prediction papers from the Dr. Chen's group is not a sufficient approach to find out which method is the best. The reason is at least two-folded: First, for each prediction paper, only a few predictors (<10 for most cases, and <5 for many) could be considered in the performance comparison, and the tools included in the comparisons also varied from paper to paper, therefore the scope of performance comparison is relatively limited. More importantly, most reports only compared the performance by cross-validation tests on the HMDD v2.0 training dataset, or included only a few case studies from literature or other databases. Therefore, the predictor performance on novel miRNA-disease associations have not been sufficiently evaluated on a sizable independent benchmarking dataset. Indeed, there was a historical reason for that, i.e., there was actually no much database resource to assemble a sizable independent benchmarking dataset before the release of our HMDD v3.0. Nevertheless, by investigating the latest papers from the predictor developers (see for example the references below [1]), we noted that there was still only cross-validation test used in the performance comparison, even the predictor was trained on the new HMDD v3.0 dataset. Therefore, a third-party benchmarking test on the sizable, independent HMDD v3.0 dataset is still necessary and important to evaluate and compare the performance of currently available miRNA-disease association predictors. 
Reference: 
[1] Wang L, You ZH, Chen X, Li YM, Dong YN, Li LP, Zheng K: LMTRDA: Using logistic model tree to predict MiRNA-disease associations by fusing multi-source information of sequences and similarities. PLoS Comput Biol 2019, 15:e1006865.* 

2. The benchmark dataset from the HMDD3.0 database is not very well for predicting new miRNA-disease associations, because the benchmark dataset includes many unreliable microRNA-disease associations that microRNA is only differentially expressed in disease compared to normal inferred from high-through technologies such as microarray. It sounds unreasonable to take unreliable data as training data to predict new microRNAs-disease associations. 

**Response: Thank you for your insightful comment and this is actually why we did this benchmarking test now on HMDD v3.2 version rather than HMDD v3.0. In HMDD v3.2, we systematically re-evaluated the causality potentials of the miRNAs to the corresponding diseases. And therefore, we could obtain a high-confidence CAUSAL benchmarking dataset. Unlike the previously used ALL benchmarking set that included all literature reported miRNA-disease associations, the CAUSAL benchmarking set only considered the miRNAs with a direct evidence of disease causing, where the weak miRNA disease associations like the microRNAs only differentially expressed in disease compared to normal were EXCLUDED. 

The benchmarking results on the high-confidence CAUSAL benchmarking set was summarized in Figure 1D and Supplementary Table S4. Briefly, although the prediction performance of all predictors is reduced on the CAUSAL benchmarking set (Figure 1D) in comparison with that on the ALL benchmarking set (Figure 1B), 9 out of the top 10 predictors were agreed between two tests (Figures 1B and 1D). These results indicate that the top predictors were able to distinguish disease miRNAs even when the low-confidence differential expressed miRNAs were excluded and only direct disease causal miRNAs were considered. Yet, the predictor developers may wish to further improve the prediction performance by considering the causality stratification provided in HMDD v3.2 database as an indicator of sample confidence, since current performance on the CAUSAL benchmarking set is still less satisfactory. We have incorporated the above results and discussion in the Page 11 of the revised manuscript.* 

3. The topic of predicting disease-related microRNAs is not very new, which has been studied for nearly ten years. Although many tools for predicting disease-related microRNAs have been published, few biologists have used these prediction tools to guide their biological experiments for verifying true miRNA-disease associations. 

**Response: We fully agreed with you that the miRNA-disease associations tools were less exploited by the biomedical researchers than expected, compared with the amount of published tools. One critical reason (in addition to the lack of benchmarking studies as mentioned previously) for that should be the fact that most of the predictors were not user-friendly to the non-specialists. We have provided an intuitive depiction of this issue in the Figure 1A: Among 90 predictors we curated from the literature, 43 predictors only provided partial prediction results (i.e. results about few diseases, usually only few common cancer types), 11 predictors do not provide result/code, or the code failed to run, and only the 36 predictors (~40%) included in this benchmarking test provided available code or prediction results. Even for these predictors, it is still quite hard for a non-specialist user who does not have much computational background to run codes written in various programming languages (Supplementary Table S6) or to query a long list of prediction results. 

In order to partially relief this issue, as also suggested by Reviewer 1#, we have incorporated the prediction results of the top predictors (including MCLPMDA, LFEMDA, LPLNS, SACMDA, ICFMDA, HLPMDA, SNMDA, LLCMDA and BLHARMDA) and the combined predictors (combining MCLPMDA, LPLNS and NAMDA) into the HMDD database. Users can either query these prediction results on the Browse page of the database or download the assembled prediction results in Excel format from the Download page of the database (http://www.cuilab.cn/static/hmdd3/data/prediction_combined.xlsx). We hope it would be helpful to increase the accessibility of current miRNA-disease prediction tools to the experimental biomedical researcher community.* 

Minor points: 
1. it may be much better that the 36 tools are categorized to evaluate the performance of each method in each category. 

**Response: Thank you for your constructive suggestions. Accordingly, we have categorized the prediction methods based on the criterion proposed by Chen et al. [2]. And the per category evaluation results was shown in Supplementary Tables S3 and S5. We have also described the top predictor from each category in the revised manuscript (please see Pages 10 and 12, respectively). 
Reference: 
[2] Chen X, Xie D, Zhao Q, You ZH: MicroRNAs and complex diseases: from experimental results to computational models. Brief Bioinform 2019, 20:515-539.* 

2. In ‘Benchmarking test and performance statistics’ section, the authors mapped all the disease terms in HMDD v2.0 and HMDD v3.0 to Disease Ontology (DO) and MeSH as benchmark set. The two ontologies have different structures, and parts of the 36 methods depend on the uniform structure of the ontology. It is confusing how to predict the novel disease-miRNA associations for those disease terms occurring only in the MeSH or DO. 

**Response: We regret for this confusion, which should be raised from our previous ambiguous description about disease term mapping method. Actually, we mapped HMDD v3.1 terms back to HMDD v2.0, so only MeSH terms were actually considered in the benchmarking test, and the disease without MeSH terms had been excluded. Therefore, the divergence of ontology structure between MeSH and DO had no influence to the benchmarking test.* 

3. The link (http://www.cuilab.cn/hmdd3/data/disease_mapping2019.txt) is not accessible. 

**Response: We feel sorry for the broken link. It is due to a typo in the previous manuscript. The correct link should be http://www.cuilab.cn/static/hmdd3/data/disease_mapping2019.txt.* 

4. The authors mentioned the prediction scores from each predicator were normalized to 0-1. It is not clear how to normalize the scores? Is it suitable for those classification-based prediction tools? 

**Response: Thank you for the constructive comment. We previously normalized the prediction scores based on the Max-Min normalization approach, which was described in more detailed in the revised manuscript (Page 24). Please note that the normalization process is only applied in the predictor combination sub-section of the manuscript, as the unified scaling of the prediction scores would facilitate further predictor combination. In order to evaluate the influence of different normalization approaches, we have performed the predictor combination with four normalization approaches: 1) Max-Min standardization, 2) Z-score standardization, 3) Sigmoid standardization and 4) no standardization. As shown in Supplementary Figure S2 (please also note that because we have optimized the predictor weights in the revised manuscript, the combination result would look different from the previous one), different normalization approaches have only marginal influence on the performance of combined predictors, so we kept the previous Max-Min normalization when performing the predictor combination in the revised manuscript.*

**Second round of review**

**Reviewer 1**

The authors have satisfactorily addressed the previous concerns. They have evaluated more prediction tools, although none of the newly assessed tools were included in the benchmark analysis based on the inclusion criteria. More significantly, the authors have incorporated the results into HMDD, which provides a useful platform for end users to easily access the data generated from this study. One minor comment: HMDD is now in Version 3.2; however, the homepage still displays V3.0.

**Reviewer 2**

The authors evaluated 36 prediction tools and gave a ranking of performance for all tools. In my opinion, this critical assessment is currently limited because there is no good benchmark dataset. The ranking of performance is only relative, and the results of these prediction tools would change greatly when somebody predicts the causal microRNAs for a disease. Authors are encouraged to choose 5 to 10 diseases, such as breast cancer, type 2 diabetes and hypertension, to check the five best predictive tools to evaluate the consistency their predictive results and performances. I still don't think the topic of predicting microRNA-disease relationships is of high priority for the high-level journals such as Genome Biology because biologists seldom experimentally validate predictions from these tools.

**Authors Response**

**Point-by-point responses to the reviewers’ comments:**

**Reviewer 1**

The authors have satisfactorily addressed the previous concerns. They have evaluated more prediction tools, although none of the newly assessed tools were included in the benchmark analysis based on the inclusion criteria. More significantly, the authors have incorporated the results into HMDD, which provides a useful platform for end users to easily access the data generated from this study. One minor comment: HMDD is now in Version 3.2; however, the homepage still displays V3.0. 

*Response: Thank you for the positive comments and constructive suggestions. We have changed the version information on the HMDD database webpage (http://www.cuilab.cn/hmdd/) to “HMDD v3.2” to address this issue.* 

**Reviewer 2**

The authors evaluated 36 prediction tools and gave a ranking of performance for all tools. In my opinion, this critical assessment is currently limited because there is no good benchmark dataset. The ranking of performance is only relative, and the results of these prediction tools would change greatly when somebody predicts the causal microRNAs for a disease. Authors are encouraged to choose 5 to 10 diseases, such as breast cancer, type 2 diabetes and hypertension, to check the five best predictive tools to evaluate the consistency their predictive results and performances. 

*Response: Thank you for the constructive comments. Indeed, the coverage of HMDD dataset is still far from perfect, and we have discussed this limitation in the revised manuscript (Pages 21-22). Nevertheless, it could already constitute a sizable benchmarking dataset (i.e. the ALL benchmarking dataset) with thousands of literature-derived miRNA-disease associations pairs. Moreover, the causal miRNA-disease associations were manually selected to assemble a high-confidence CAUSAL benchmarking dataset with improved quality. As already shown in our previous responses, 9 out of the top 10 predictors were shared between the results on the ALL benchmarking datasets and those on the CAUSAL benchmarking datasets, indicating good consistency of the benchmarking results in predicting causal miRNA-disease associations.* 

*According to your helpful suggestions, we also performed the per disease evaluation for 9 common diseases (including but not limited to breast cancer, type 2 diabetes and hypertension). The results were summarized in Supplementary Figure S1 and discussed in Page 10 of the revised manuscript. On the one hand, the predictors showing the best overall prediction performance would still rank in the top five for most (7 out of 9) common diseases, suggesting the consistency of the performance ranking. On the other hand, some predictors including NSEMDA, RFMDA, PRMDA, BNPMDA and MCMDA were also highlighted because of their superior accuracy for some common diseases.* 

I still don't think the topic of predicting microRNA-disease relationships is of high priority for the high-level journals such as Genome Biology because biologists seldom experimentally validate predictions from these tools. 

*Response: In our opinion, the meaning of “experimentally validate” would be two-folded: 1) It concerns the question that whether the prediction results can be confirmed by further experiments. This question has not been systematically investigated with a sizable, independent benchmarking test, and our analysis clearly showed that at least the top predictors can be validated by the subsequently published experimental results with acceptable accuracies (Figure 1). 2) It concerns whether the biomedical researchers intentionally take the prediction results as the targets for future experimental validation. As we have already stated in our previous response, although our benchmarking results have suggested the merits of current miRNA-disease predictors, the availability of these predictors have limited the impact of these tools among the biomedical researcher community. Besides, a comprehensive benchmark of these predictors is also important for biomedical researchers to choose adequate predictors for their own purpose. As one step to improve these two outstanding issues, here we have incorporated the prediction results of the top predictors and those of the combined predictor to our public miRNA-disease association database HMDD (http://www.cuilab.cn/hmdd/), in order to increase the accessibility of the miRNA-disease predictors to the biomedical research community.*
